# ATP-sensitive peptide-based coacervates for intracellular delivery of therapeutic oligonucleotides

**DOI:** 10.3389/fmolb.2026.1767656

**Published:** 2026-02-13

**Authors:** Tatiana Vedekhina, Vladislav Anufriev, Nicole Polischuk, Elizaveta Malakhova, Sabina Alieva, Viacheslav Severov, Margarita Bogomiakova, Pavel Bobrovsky, Anna Varizhuk

**Affiliations:** 1 Lopukhin Federal Research and Clinical Center of Physical-Chemical Medicine of Federal Medical Biological Agency, Moscow, Russia; 2 MIREA–Russian Technological University, Moscow, Russia; 3 University of Vienna, Vienna, Austria; 4 Lomonosov Moscow State University, Moscow, Russia

**Keywords:** ATP, coacervates, drug delivery, peptide vehicles, therapeutic nucleic acids

## Abstract

Despite advances in the fields of lipoplexes, metal nanoparticles, and other nucleic acid carriers, intracellular delivery of DNA/RNA therapeutics remains a pressing area of molecular medicine. The existing delivery systems have limitations in terms of stability, efficacy, or toxicity. Peptide-based coacervates have recently emerged as a promising alternative. They offer several advantages, including easy DNA/RNA incorporation, low toxicity, and the ability to penetrate membranes. However, they have one main drawback: inefficient intracellular unpacking. In this study, we present a novel approach to programmed drug release from peptide-based coacervates. We used a previously described intrinsically disordered histidine-rich peptide as a coacervate scaffold and introduced a viral or human protein-derived ATP-responsive module into its sequence. We assembled the coacervates by mixing the resulting peptides with RNA and a model oligonucleotide (ODN), plasmid DNA, or mRNA in a pseudophysiological buffer. The admixtures of labeled peptides and ODN enabled monitoring coacervate formation and dynamics using fluorescence microscopy. The coacervates were relatively stable in ATP-free environments and underwent rearrangements involving the partial release of the ODN in the presence of ATP. The coacervates penetrated HEK293 cells within 4 hours and released the ODN into the cytoplasm within 20 h. They were inferior to the ATP-insensitive (control) peptide in delivery assays with plasmid DNA and mRNA but outperformed the control peptide in assays with the ODN. Our preliminary results suggest that ATP-sensitive coacervates have potential as ODN carriers.

## 1 Introduction

Efficient intracellular delivery of nucleic acid (NA) fragments and derivatives is important for fundamental research and therapeutic strategies involving the use of antisense oligonucleotides, synthetic small interfering RNA, guide RNA, etc., for mRNA targeting or gene editing (Sabatos-Peyton et al., 2010; Vickery et al., 2011; Bartolucci et al., 2022). Liposomal, polymeric and micellar nanoparticles are commonly used as delivery vehicles for mRNA or antisense oligonucleotides (Lorscheider et al., 2021; Pacheco et al., 2023). Their limitations include the lack of biodegradation, low bioavailability toxicity, etc. (Chong et al., 2014; Gupta et al., 2019; Kumawat et al., 2019; Lu et al., 2020; Wang et al., 2022).

Coacervate-based delivery systems are a promising modern alternative to classical (liposomal or polymeric) nanoparticles. NA incorporation occurs throughout the coacervate volume due to multiple weak electrostatic interactions and is characterized by high efficiency. Furthermore, coacervates effectively protect therapeutic NAs from biodegradation (Sun et al., 2022). The main advantages of coacervates over classical nanoparticles include biocompatibility, ease of cell penetration, and low cytotoxicity (Jo et al., 2019; Xiao et al., 2020). In addition to delivering NAs or low-molecular-weight agents to tumor cells (Liu et al., 2022), coacervates and similar hydrogel-type systems have been used to deliver protein growth factors to damaged tissues (Kim et al., 2018; Wu et al., 2018). These applications demonstrate the potential of coacervates in regenerative medicine, oncotherapy, and allergy treatment.

Of the peptide-based coacervates being considered as delivery systems, HB*pep* coacervates are the most thoroughly characterized (Lim et al., 2018; 2020; Shebanova et al., 2022; Sun et al., 2022; 2025; Liu et al., 2023; Pramanik et al., 2025). The HB*pep* sequence (GHGVY GHGVY GHGPY GHGPY GHGLY W), derived from the squid beak protein HBP (Tan et al., 2015), shows high propensity for coacervation due to multiple His and Gly residues (Blocher and Perry, 2017). The coacervates successfully delivered therapeutic agents into cells but remained stable for several days, resulting in inefficient drug release (Lim et al., 2018). To solve this problem, Sun et al. modified the peptide design to make it redox-sensitive (Sun et al., 2022). Specifically, they inserted a single Lys residue into HB*pep*, which shifted the optimal pH for coacervation from 7.5 to 9.0, and masked this Lys residue with a redox-sensitive (disulfide bond-containing) “self-destructive” moiety. The resulting HB*pep* variant (HB*pep*-SR) formed coacervates that readily incorporated guest macromolecules at pH 6.5 and released them after crossing the cellular membrane in response to intracellular glutathione. The only drawback of this approach is the complexity of HB*pep*-SR synthesis.

Here, we report a different approach to fine-tuning coacervates for efficient drug release. To facilitate intracellular disassembly, we introduced an ATP-sensitive module into the HB*pep* sequence. This modification is compatible with the standard peptide synthesis and thus might be more convenient than HB*pep*-SR. We report the selection of an optimal ATP-sensitive module and verification of its effect on the coacervate packing and unpacking.

## 2 Materials and methods

### 2.1 Peptides, oligonucleotide, plasmids, and mRNA

Oligodeoxyribonucleotide (ODN) 22AG (purity >95%, HPLC), labeled with dichloro-diphenyl-fluorescein (SIMA) was obtained from Litekh (Russia).

pCMV-Fluc, the PGL4-based plasmid with the Firefly luciferase (FLuc) gene under the cytomegalovirus (CMV) promoter, was obtained from Promega (United States).

pEGFP-N1(GenBank Accession #U55762), the pCDNA3.1-based plasmid with the enhanced green fluorescence protein (GFP) gene under the CMV promoter, was obtained from CLONOTECH Laboratories, Inc. (United States).

Torula yeast-derived mixed-sequence RNA (random RNA) was purchased from HiMedia Laboratories (Thane, India).

mRNA Fluc was prepared as follows. To obtain a DNA template for *in vitro* transcription, we designed a construct encoding Fluc with beta-globin 3′-and 5′-UTRs, which have been proposed previously for mRNA vaccines (Mueller and Fine, 2021), under the T7 promoter:

TAATACGACTCACTATAG*AATAAACTAGTATTCTTCTGGTCCCCACAGACTCAGAGAGAACCCGCCACC*ATGN_x_TGATGA*GCTGCCTTCTGCGGGGCTTGCCTTCTGGCCATGCCCTTCTTCTCTCCCTTGCACCTGTACCTCTTGGTCTTTGAATAAAGCCTGAGTAGGAAGGCGGCCGC*

Bold, T7 promoter; Italics, UTRs; underlined, start/stop codones, N_x_ – the Fluc coding sequence. The Fluc fragment was amplified from pCMV-Fluc, and other fragments were assembled from synthetic ODNs.

The T7-FLuc construct was cloned into the pCR2.1 vector (Invitrogen, United States). The resulting plasmid was transformed into *E. coli* Top10 competent cells, and the cells were grown overnight on an ampicillin-containing agar at 37 °C. Colony PCR screening was performed using M13F/M13R primers. Selected clones were incubated overnight. Then, the plasmid was isolated using the Plasmid Midi Kit (Qualigen, United States), and its sequence was verified by Sanger sequencing.

*In vitro* transcription was performed using the “*In Vitro* mRNA Synthesis Kit (with m7GmAmG)” kit (Biolabmix, Russia), according to the manufacturer’s instructions, substituting ΨTP for UTP. The resulting RNA was isolated using the DR-50 kit for DNA and RNA isolation from reaction mixtures. Polyadenylation was then performed using the mRNA Poly(A) Tailing Kit (Invitrogen, United States), following to the manufacturer’s protocol. The transcript length before polyadenylation was verified by electrophoresis in 1% agarose.

Peptides were synthesized using the Fmoc strategy and standard protocols for the solid-phase peptide synthesis with microwave treatment of the reaction mixture (Vedekhina et al., 2024) on a Liberty Blue Synthesizer (CEM Corporation, Matthews, United States).

### 2.2 Coacervate assembly, ATP-sensitivity assays, and microscopy imaging

To assemble the coacervates, a water solution of the HB*pep*-ATP peptide (99% unlabeled and 1% FITC-labeled) was supplemented with random RNA and SIMA-22AG in a 10 mM Na-phosphate buffer (pH 7.4) containing 150 mM NaCl, achieving final concentrations of 5 mg/mL (peptide), 3 mg/mL (RNA) and 10 μM (ODN). We assessed the sensitivity of the peptide coacervates to ATP by measuring the number of coacervates and their size as at different ATP concentrations (2.5–20 mM). Coacervates were visualized using an Eclipse Ti2 microscope (Nikon, Japan) with FITC excitation at 488 nm and a cut-off filter of 520 nm or with SIMA excitation in the 531 nm and a cut-off filter of 555 nm. ImageJ (Fiji) software, version 1.54d, was used to analyze the colocalization of FITC-peptides and SIMA-22AG. The DropletCalc program (Shtork et al., 2025) was used to estimate the radii and the total area of the coacervate projections S_t_, which, in the first approximation, can be linked to coacervate number *N* and the average radius r_av_: S_t_ ∼ *N*πr_av_^2^. The S_t_ values were normalized by the area of the glass support, and the resulting normalized values (%) are indicative of the coacervate fraction.

The efficiency of SIMA-22AG loading into coacervates was calculated as a percentage of SIMA-positive (red) pixels within FITC-positive (green) droplets normalized by the mean droplet:intial solution SIMA intensity ratio. Relative SIMA intensities and the pixel fraction were calculated using the ImageJ analysis tools (Fiji software, version 1.54d).

### 2.3 Fluorescence recovery after photobleaching (FRAP)

FRAP experiments were performed using an Olympus FV3000 microscope equipped with a ×40 objective lens. The FITC-labeled peptide HB*pep*-ATP-1 in the coacervate was excited using an argon laser at 488 nm, and the emission signals were collected within the 500–530 nm range. HB*pep*-ATP-1 photobleaching was performed using a 10x pulse of a laser (488 nm) at 10% intensity for the FITC-peptide (40 s) through half of the coacervate (10–12 μm). After photobleaching, images were acquired at a rate of 3.2 s per frame and at 1% intensity every min during the first 10–15 min and then every 5 min. The fluorescence intensity of the bleached area over time was calculated using ImageJ (Fiji software, version 1.54d) and normalized by the intensity of the nearby non-bleached droplet.

### 2.4 Cytotoxicity assays

The viability of coacervate-treated HEK-293 cells was assessed by using the EZBlue™ Cell Assay Kit (HiMedia Laboratories, India). HEK-293 cells were cultured in a humidified incubator at 5% CO_2_ and 37 °C, tested for *mycoplasma* infection, and grown in a 96-well plate in DMEM (PanEco, Russia) supplemented with 1% penicillin/streptomycin (PanEco, Russia), 2 mM glutamine (PanEco, Russia), and 10% FBS (HyClone GE Healthcare, United States).

After incubating for 24 h at 37 °C with 5% CO_2_, 100 µL of medium were removed from each well. Then, 100 µL of freshly prepared suspensions of HB*pep*-ATP peptide and random RNA coacervates at various concentrations (from 0.04 to 2.5 mg/mL of peptide, respectively) were added to the medium. After 2 h of incubation at 37 °C, 10 μL of EZBlue™ Solution (HiMedia Laboratories, India) was added to the each well, and the cells were incubated for 2 more hours. Fluorescence was then measured at 560 nm excitation (EX) and590 nm emission (EM) 4 and 24 h after adding the peptides using an Infinite M200 PRO plate reader (Tecan, Switzerland).

### 2.5 Coacervate-based transfection and verification of its efficiency

HEK-293 cells were grown in a 96-well plate as described above to a confluency of ∼60%. For SIMA-22AG delivery,100 μL of freshly prepared suspensions of oligonucleotide-loaded peptide coacervates (2 μM oligonucleotide, 1 mg/mL HB*pep*-ATP peptides, 0.5 mg/mL random RNA) were added to each well after removing the medium. After 4 h of incubation, the solution was removed, and the cells were washed twice with PBS. Then 100 μL of fresh medium (DMEM, 10% FBS, antibiotics) was added, and the cells were incubated for an additional 20 h at 37 °C. Penetration and unpacking of the coacervates were monitored using the JuLI™ Stage imaging system (NanoEntek, South Korea).

To deliver EGFP- or Fluc-encoding plasmid DNAs (pDNAs) or the Fluc-encoding mRNA, DMEM was replaced with 90 μL of OptiMEM. Next, 10 μL of freshly prepared pDNA- or mRNA-loaded peptide coacervates (0.5 mg/mL HB*pep*-ATP variants, 0.3 mg/mL RNA, 10 μg/mL pDNA or mRNA) were added. Lipofectamine™ and MessengerMAX™ (Invitrogen, United States) were used as control transfection reagents, and the respective procedures were performed according to the manufacturer’s protocols. After 4 h of incubation, the transfection solutions were replaced with fresh medium, and the cells were incubated for additional 24 h (EGFP) or 48 h (Fluc). The EGFP fluorescent signal was detected using the JuLI™ Stage imaging system (NanoEntek, South Korea). Fluc transfection was assessed using the One-Glow Luciferase Assay System (Promega, United States), following the manufacturer’s protocol. The luciferase luminescent signal was measured using an Infinite M200 PRO plate reader (Tecan, Switzerland).

## 3 Results and discussion

We aimed to obtain HB*pep*-based peptides that assemble into ATP-sensitive coacervates for ATP-driven intracellular disassembly. The ability of ATP to solubilize peptides and proteins is due to its amphiphilic nature. The combination of a charged fragment (phosphate) and an aromatic fragment (nucleobase) enables ATP to disrupt typical coacervate contacts, including π-π/π-cation interactions between aromatic residues and the cationic amino acid residues, as well as ionic bonds, van der Waals interactions, and hydrogen bonds (Nott et al., 2015; Riback et al., 2017; Vernon et al., 2018; Chiu et al., 2020).

We searched for short ATP-binding motifs to introduce into the HB*pep* sequence and selected consensus motifs that are overrepresented in human kinases (Xiao et al., 2013) and fragments of viral proteins (Mujeeb et al., 1994; Titolo et al., 1999; Scholz, 2003). Motif 1 (the P-loop sequence GxxxxGK) was identified by screening biotin-labeled peptides from all human ATP-binding proteins, and motif 2 (HRDxKxxN) was derived exclusively from kinases (Xiao et al., 2013). The viral motifs include the ATP-binding sites of the terminase subunit pUL56 of the human cytomegalovirus (Scholz, 2003) (motifs 3, 4, and 5) and the helicase of the human papillomavirus type 11 E1 (Titolo et al., 1999) (motif 6). Table 1 shows the sequences of the resulting peptides with the ATP-binding modules (HB*pep*-ATP-1–6), as well as the control peptide lacking the ATP module (HB*pep*-contr). Initially, we added each ATP-sensitive module to the C-terminus of the peptide (HB*pep*-ATP-1–6). After the first round of assays, we selected a leader, and added its analog with the ATP-module shifted to the N-terminus (HB*pep*-ATP-1Nt) to the peptide set.

**TABLE 1:** Sequences of HB*pep*-ATP variants.

Code	Sequence	ATP-module	References
HB*pep*-ATP-1	GHGVY GHGVY GKGGFGKVTLVRK GHGPY GHGLYW	human (motif 1)	Xiao et al. (2013)
HB*pep*-ATP-2	GHGVY GHGVY IIHRDLKPENILL GHGPY GHGLYW	human (motif 2)	Xiao et al. (2013)
HB*pep*-ATP-3	GHGVY GHGVY RARGGGKK GHGPY GHGLYW	viral (motif 3)	Scholz (2003)
HB*pep*-ATP-4	GHGVY GHGVY YNETFGKQ GHGPY GHGLYW	viral (motif 4)	Scholz (2003)
HB*pep*-ATP-5	GHGVY GHGVY YNETFGKQ RARGGGKK GHGPY GHGLYW	viral (motif 5)	Scholz (2003)
HB*pep*-ATP-6	GHGVY GHGVY GPPNTGKS AALVDD VTSNI GHGPY GHGLYW	viral (motif 6)	Titolo et al. (1999)
HB*pep*-ATP-1Nt	GKGGFGKVTLVRK GHGVY GHGVY GHGPY GHGLYW	human (motif 1)	Xiao et al. (2013)
HB*pep*-contr	GHGVY GHGVY GHGPY GHGPY GHGPY GHGLYW	-	-

Underlined words represents the ATP-binding modules.

**FIGURE 1:**
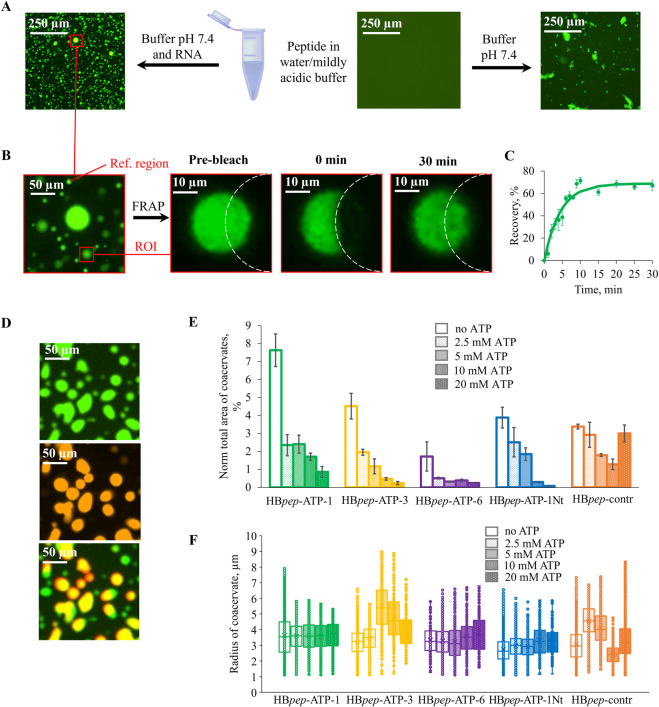
Biophysical characterization of HB*pep*-ATP variants. **(A)** Conditions for coacervation of HB*pep*-ATPs. **(B,C)** Fluorescence micrographs **(B)** and recovery curves **(C)** of HB*pep*-ATP-1. Data are presented as the mean ± SD of n = 3 independent experiments. **(D)** Colocalization of FITC-HB*pep*-ATP-1 (green) and SIMA-22AG (red). **(E)** Fractions of coacervates, calculated based on their total projection area, at different ATP concentrations. **(F)** Coacervate size at different of ATP concentrations.

To facilitate imaging of the coacervates, the peptides were labeled at their N-termini with a fluorescein isothiocyanate (FITC) residue, which fluoresces in the green range of the spectrum. The labeled peptides were then mixed 1:99 with the unlabeled peptides. Random RNA was added to stimulate coacervation. In line with previous reports for HB*pep* (Harrison and Shorter, 2017), peptide coacervation was inefficient in weakly acidic aqueous solutions and in the absence of RNA (Figure 1A). After adding RNA in a pH 7.4 buffer, peptides HB*pep*-ATP-1(Nt), HB*pep*-ATP-3, and HB*pep*-ATP-6 (Supplementary Figures S1, S2) exhibited good coacervation, similar to HB*pep*-ATP-contr (Supplementary Figures S3). The resulting coacervates appeared as brightly colored, mobile, micrometer-sized droplets that partially wetted the glass surface (Figure 1A). Their circularity was ≥0.8. The coacervates were stable in ATP-free solutions for at least 24 h. HB*pep*-ATP-2 separated with RNA, but some of the resulting droplets had irregular shapes (circularity ≤0.6), which suggests gelation or solidification. In the cases of HB*pep*-ATP-4 and HB*pep*-ATP-5, phase separation was minor (Supplementary Figure S4). We conclude that ATP-sensitive motifs 2, 4, and 5, but not motifs 1, 3, and 6, hamper droplet formation.

The liquid state of the observed droplets was confirmed using fluorescence recovery after photobleaching (FRAP) (Figures 1B,C). A fragment of a droplet from the FITC-labeled HB*pep*-ATP-1-containing sample was bleached, and the fluorescence recovery was monitored for 30 min. Representative images are shown in Figure 1B. The recovery reached ≈65% (Figure 1C) and was moderately slow, with an apparent tau_1/2_ of 190 ± 30 s, which is consistent with the dense liquid state. Therefore, the observed droplets are indeed coacervates rather than solid aggregates.

To verify the ability of coacervates to incorporate nucleic acid therapeutics, we used a G-quadruplex-forming fragment of human telomeric repeats (GGGTTA)_3_GGG (22AG) as a model ODN. Quadruplexes have recently emerged as a promising new type of anticancer drug candidates. The first FDA-approved therapeutic of this type (World Health Organization, 2024), Imetelstat (RYTELO™), is a lipid-conjugated oligonucleotide (Carloni et al., 2021; Keam, 2024) that inhibits telomerase and can be regarded as a 22AG mimic.

The SIMA-labeled model ODN (SIMA-22AG), that fluoresces in the red channel, was added to a mixture of unlabeled and FITC-labeled peptides at pH 7.4. This had no significant effect on the morphology of the coacervates visualized in the green channel (Supplementary Figures S1-S3). The colocalization of coacervates in the red and green channels (Figure 1D) confirms the ODN incorporation. A few ODN-free droplets were observed, and the colocalization seemed to be incomplete for small droplets, likely due to their rapid movement revealed by visual screening. Nevertheless, the apparent colocalization was within the range of 75%–90% for all HB*pep* variants, and the ODN loading efficiency (60% ± 20% for HB*pep*-ATP-1, 40% ± 3% for HB*pep*-ATP-3, 15% ± 2% for HB*pep*-ATP-6, 38% ± 9% for HB*pep*-ATP-1Nt, and 40% ± 10% for HB*pep*-ATP-contr) varied in accordance with the total coacervate projection area (Figure 1E).

Next, we tested the efficiency of ATP-driven coacervate unpacking *in vitro* at ATP concentrations ranging from 2.5 to 20 mM. Coacervates of all peptides, except HB*pep*-contr, with random RNA and SIMA-labeled ODN showed clear, dose-dependent disassembly (Supplementary Figure S1-S3). It was evident from the analysis of the total coacervate projection area (Figure 1E). In contrast, the ATP-dependence of HB*pep*-contr coacervates was irregular and the least pronounced (Figure 1E; Supplementary Figure S3). HB*pep*-ATP-3 coacervates also exhibited an ATP-dependent decrease in average coacervate size (Figure 1F; Supplementary Figure S2).

**FIGURE 2:**
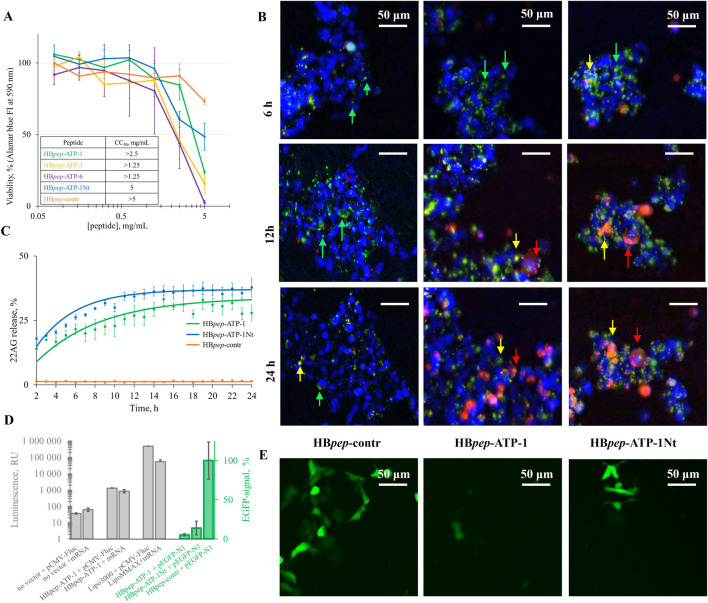
Cytotoxicity of HB*pep*-ATPs and intracellular delivery of different types of nucleic acids. **(A)** Cytotoxicity of HB*pep*-ATPs on HEK293 cells. **(B)** Intracellular delivery of SIMA-22AG: representative fluorescence microscopy images. Arrows mark coacervates in different states: green, intact coacervates; yellow, the disassembly begins (the cargo label is visible but remains mostly colocalized with the peptide label); red, the cargo is released. **(C)** Kinetic curves summarizing the release of SIMA-22AG from HB*pep*-ATP-1/HB*pep*-ATP-1Nt/HB*pep*-contr in HEK-293. The curves were normalized by the ODN loading efficiency. **(D)** Intracellular delivery of luciferase-encoding mRNA and luciferase-encoding plasmid pCMV-Fluc evaluated by using luciferase assays (gray) and the delivery of EGFP-encoding plasmid pEGFP-N1 evaluated by fluorescence microscopy (green). **(E)** Representative fluorescence microscopy images of HEK-293 cells transfected with pEGFP-N1 within HB*pep*-contr, HB*pep*-ATP-1, and HB*pep*-ATP-1Nt coacervates (the images were taken 24 h after transfection).

Before testing the coacervates as intracellular delivery vehicles, we evaluated their cytotoxicity. Figure 2A summarizes their effects on the viability of HEK293 cells. Most of the coacervates were nontoxic at concentrations up to 1 mg/mL, with CC_50_ values exceeding 1.25 mg/mL in all cases.

Next, we tested the coacervate-based delivery of the model oligonucleotide 22AG to HEK293 cells. We monitored the internalization of FITC-labeled HB*pep*-ATP-based coacervates (green) and SIMA-labeled 22AG (red) using fluorescence microscopy in real time (Figure 2B; Supplementary Figures S5-S7). The resulting kinetic curves are shown in Figure 2C. HB*pep*-ATP-3- and HB*pep*-ATP-6-based coacervates either failed to enter cells or were rather unstable in the biological media (despite the reasonable stability in PBS) and disassembled prior to cellular uptake. HB*pep*-contr-based coacervates entered cells but remained stable after the uptake for 24 h and showed inefficient 22AG release (Supplementary Figure S5). HB*pep*-ATP-1-based coacervates entered cells within 4 h and released SIMA-22AG into the cytoplasm within 20 h (Supplementary Figure S6). Thus, HB*pep*-ATP-1 was the most promising peptide from the initial set with the ATP module at the C-terminus. Because the position of the module can affect the peptide’s physical properties and performance in delivery assays, we obtained an HB*pep*-ATP-1 analog, HB*pep*-ATP-1Nt, with the same kinase-derived ATP module at the N-terminus, and repeated all the experiments. The performance of HB*pep*-ATP-1Nt *in vitro* was mostly similar to that of HB*pep*-ATP-1 (Figures 1D,E; Supplementary Figure S1), while in HEK-293 cells it slightly outperformed HB*pep*-ATP-1 in oligonucleotide delivery assays (Figures 2B,C; Supplementary Figures S6, S7).

Finally, we examined the potential use of the new coacervates in delivering long therapeutic nucleic acids, such as mRNA or DNA, which are being used more frequently as vaccine components. To do so, we used a model mRNA that encodes the *Firefly* luciferase reporter protein (FLuc), a model plasmid that contains the same reporter gene (pCMV-Fluc), and a plasmid that encodes green fluorescent protein (pEGFP-N1). We assessed the efficiency of mRNA/pCMV-Fluc delivery to HEK293 cells in HB*pep*-ATP-1 coacervates using standard luciferase assays, and the efficiency of pEGFP-N1 delivery in HB*pep*-ATP-1, HB*pep*-ATP-1Nt, or HB*pep*-contr coacervates using fluorescence microscopy imaging 1 day after transfection (Figure 2D). For the control assays, we used standard transfection reagents Messenger Max and Lipofectamine 3,000 for mRNA and DNA, respectively. HB*pep*-ATP-1 substantially underperformed to the known transfectants in Fluc assays and was inferior to HB*pep*-contr in pEGFP delivery assays. HB*pep*-ATP-1Nt slightly outperformed HB*pep*-ATP-1 in the pEGFP assays but was still substantially inferior to HB*pep*-contr.

To gain insight into possible reasons for the poor performance of HBpep-ATP-1 in the plasmid delivery assay compared to the ODN delivery assay, we performed additional FRAP experiments with 22AG-loaded and pEGFP-loaded coacervates (Supplementary Figure S8) and compared the results to those obtained for the cargo-free coacervates (Figure 1B). ODN 22AG caused no substantial changes in the recovery kinetics of FITC-HBpep-ATP-1 (tau_1/2_ = 220 ± 20 s), whereas pEGFP slowed it down significantly (tau_1/2_ = 440 ± 30 s). The latter result suggests reduced diffusion within coacervates, which suggests reduced DNA release and may partly explain reduced expression.

Thus, HB*pep*-contr is suitable for the intracellular delivery of the lengthy DNA/RNA, probably because such a cargo can be released in an ATP-independent manner. In contrast, the ATP module-containing peptides HB*pep*-ATP-1 and HB*pep*-ATP-1Nt are tuned for short oligonucleotides. Lengthy nucleic acids can form multiple transient interactions with the ATP modules, yielding coacervates of increased density, which may account for inefficient unpacking. Thus, future studies of the coacervates could include determination of the threshold nucleic acid size and the optimization of the peptide:nucleic acid ratio. Additionally, the mixed-sequence random RNA could be substituted for a more appropriate homogeneous biopolymer.

To summarize, we have demonstrated that HB*pep*-ATPs form coacervates that incorporate different NAs. These cargo-loaded coacervates are engulfed by HEK293 cells and disassemble, supposedly due to the relatively high intracellular ATP levels (0.2–15.8 mM (Greiner and Glonek, 2021)), releasing the cargo into the cytosol. The precise uptake mechanism of such peptide coacervates awaits clarification. Based on the previous studies of other coacervates, lipid-raft-mediated endocytosis appears to be the likely one (Vedekhina et al., 2025). The coacervates based on HB*pep* variants with the human kinase-derived ATP module were superior to unmodified HB*pep* in assays with the model ODN 22AG, making them promising platforms for intracellular delivery of short oligonucleotide therapeutics.

## Data Availability

The original contributions presented in the study are included in the article/Supplementary Material, further inquiries can be directed to the corresponding author.
