# Supplementary material for: ATP-sensitive peptide-based coacervates for intracellular delivery of therapeutic oligonucleotides
Source: Front Mol Biosci. 2026 Feb 13;13:1767656. doi: 10.3389/fmolb.2026.1767656 (PMC12945794; doi:10.3389/fmolb.2026.1767656)
Supplement: Supplementary file 1 [file Supplementaryfile1.docx]

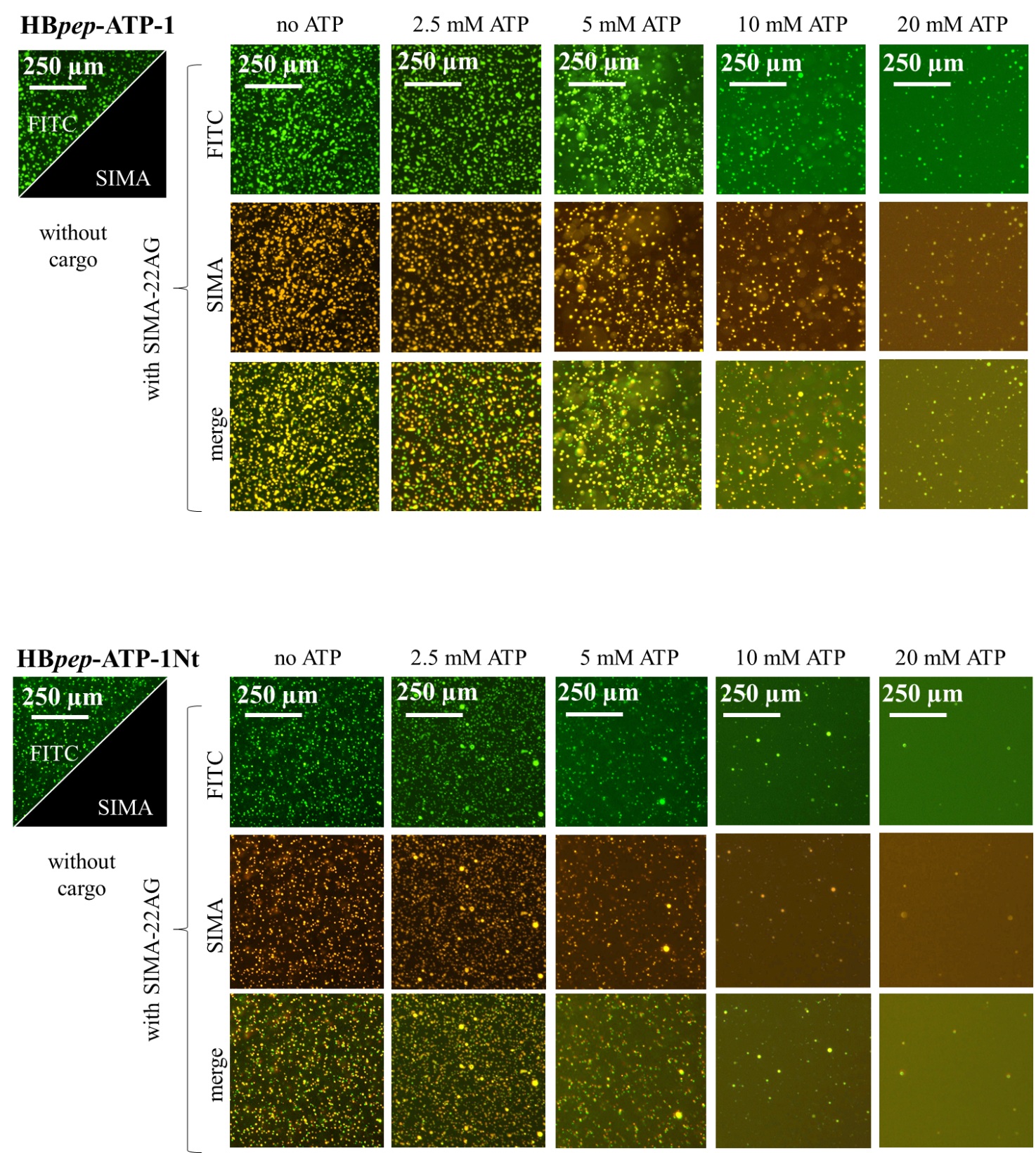


**Figure S1.** Phase separation of HB*pep*-ATP-1 and HB*pep*-ATP-1Nt: fluorescence micrographs o the coacervates obtained in the absence of the nucleic acid cargo (left) and in the presence SIMA-22AG with increasing ATP concentrations (right).


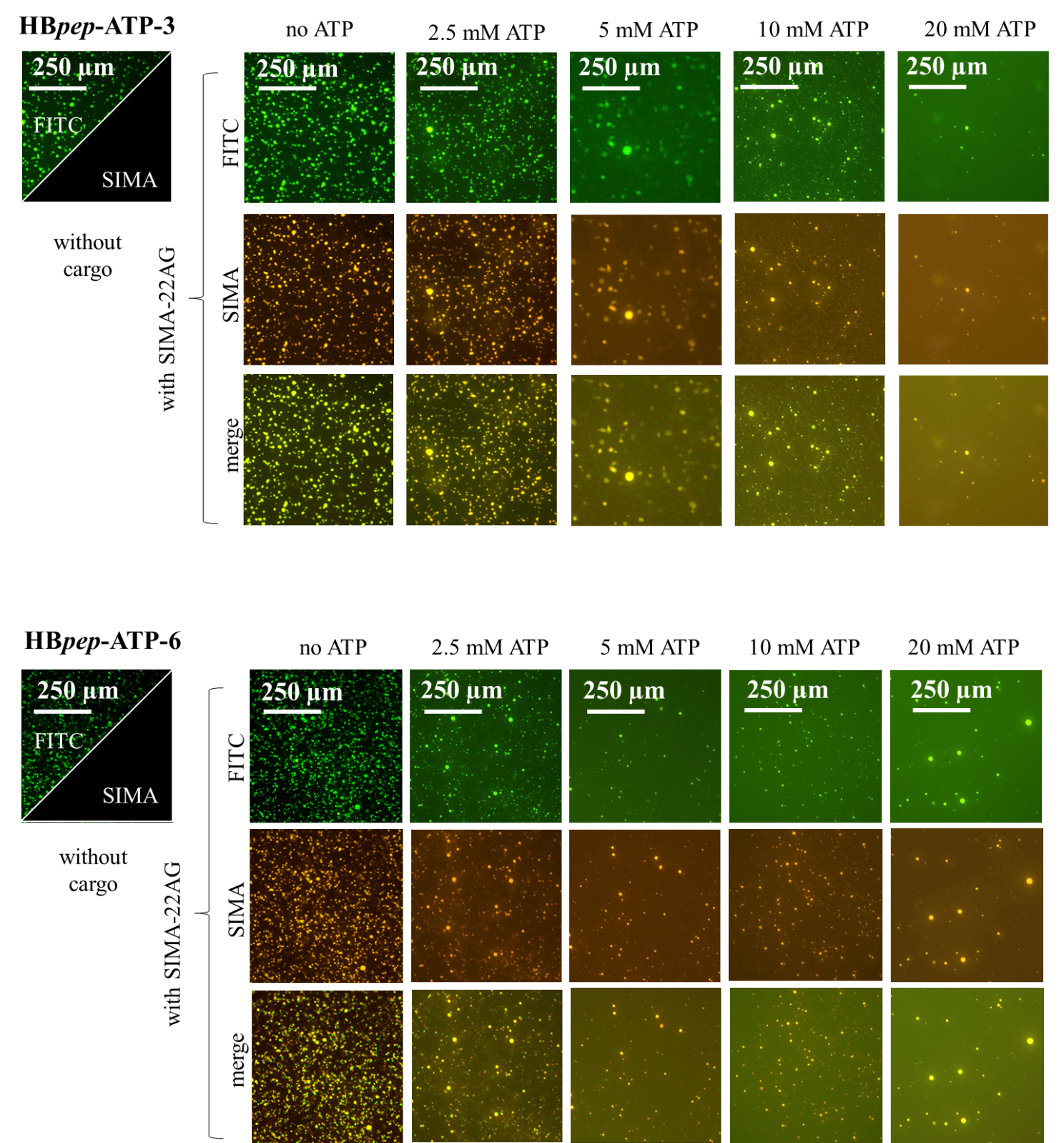


**Figure S2.** Phase separation of HB*pep*-ATP-3 and HB*pep*-ATP-6: fluorescence micrographs o the coacervates obtained in the absence of the nucleic acid cargo (left) and in the presence SIMA-22AG with increasing ATP concentrations (right).


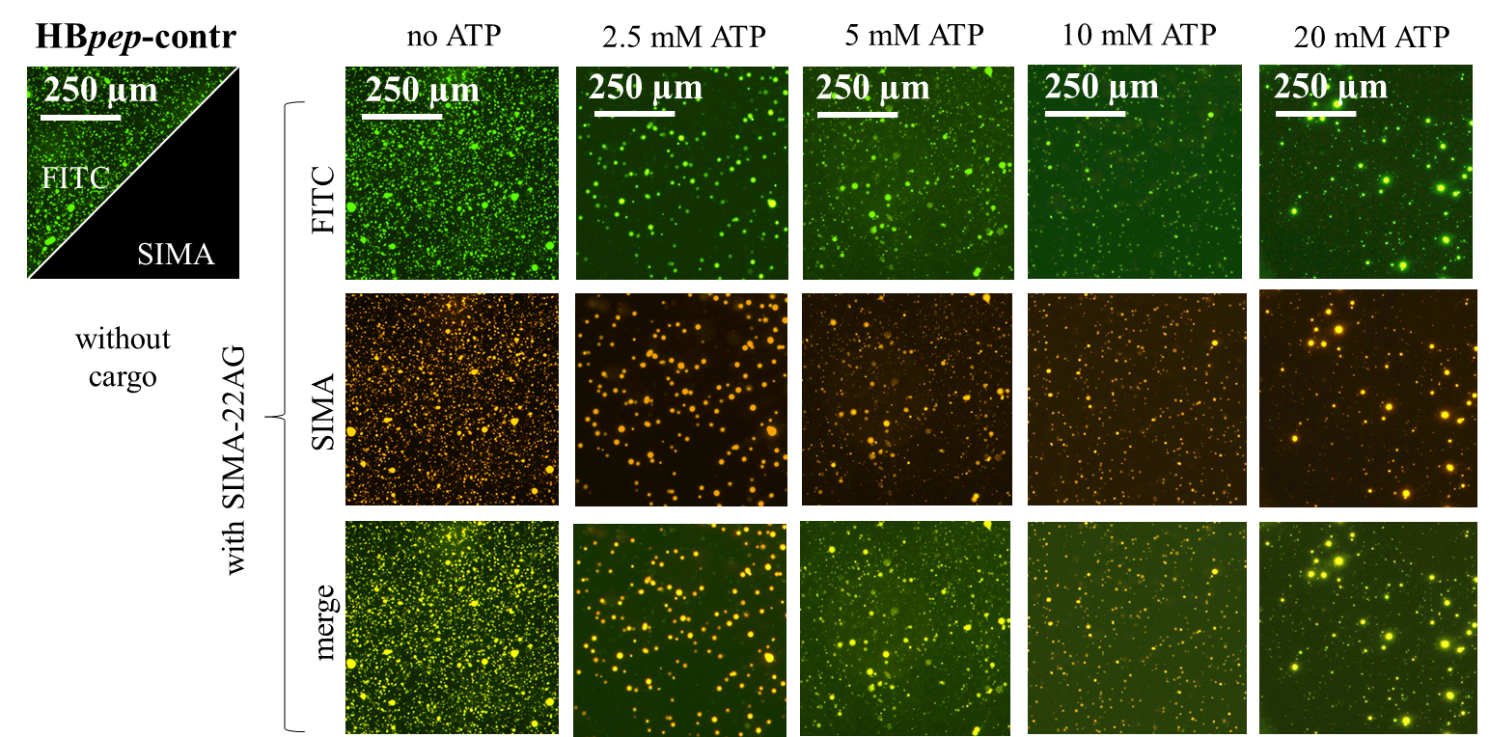


**Figure S3.** Phase separation of HB*pep*-contr: fluorescence micrographs o the coacervates obtained in the absence of the nucleic acid cargo (left) and in the presence SIMA-22AG with increasing ATP concentrations (right).


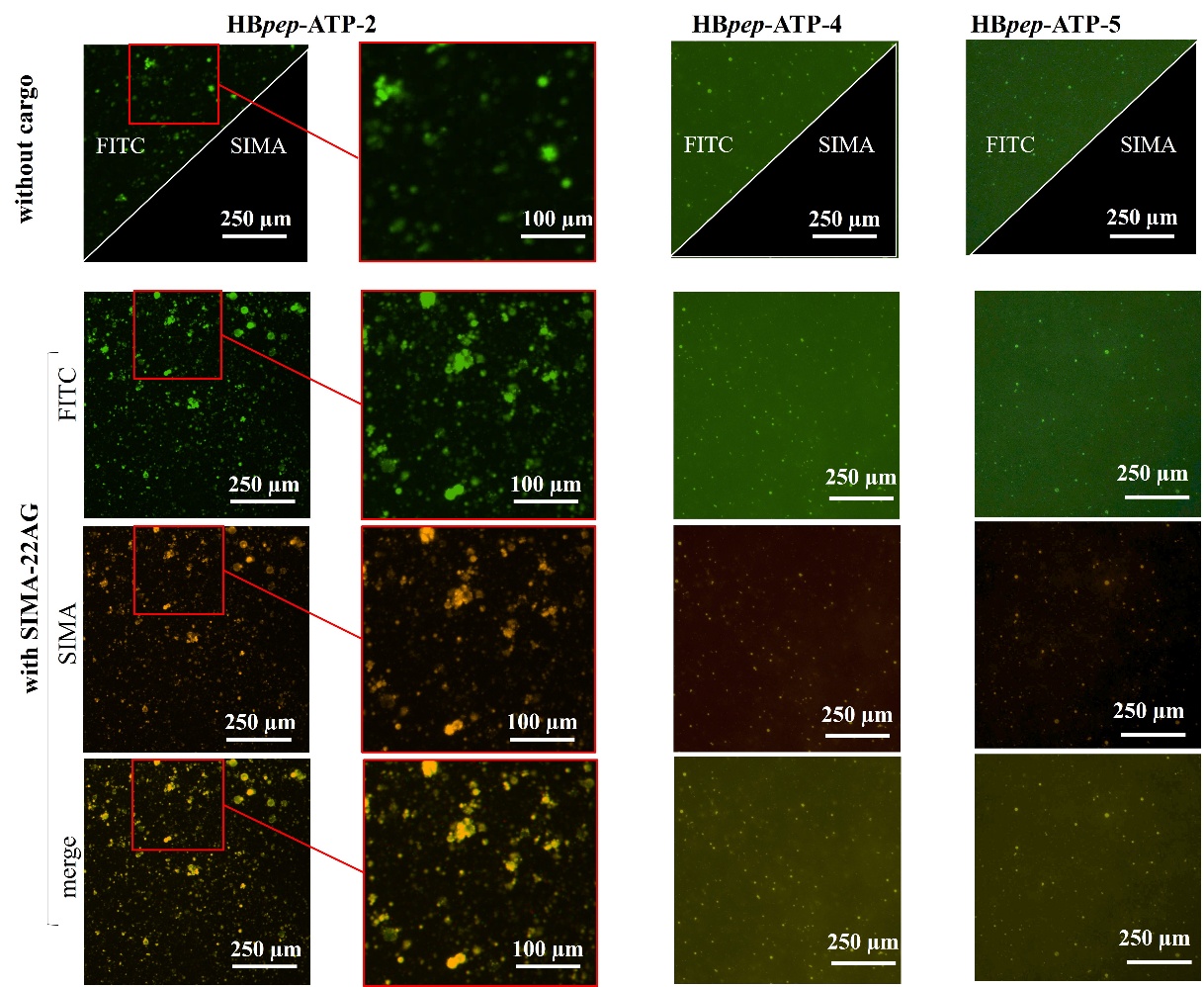


**Figure S4.** Gelation of HB*pep*-ATP-2 and minor phase separation of HB*pep*-ATP-4,5.


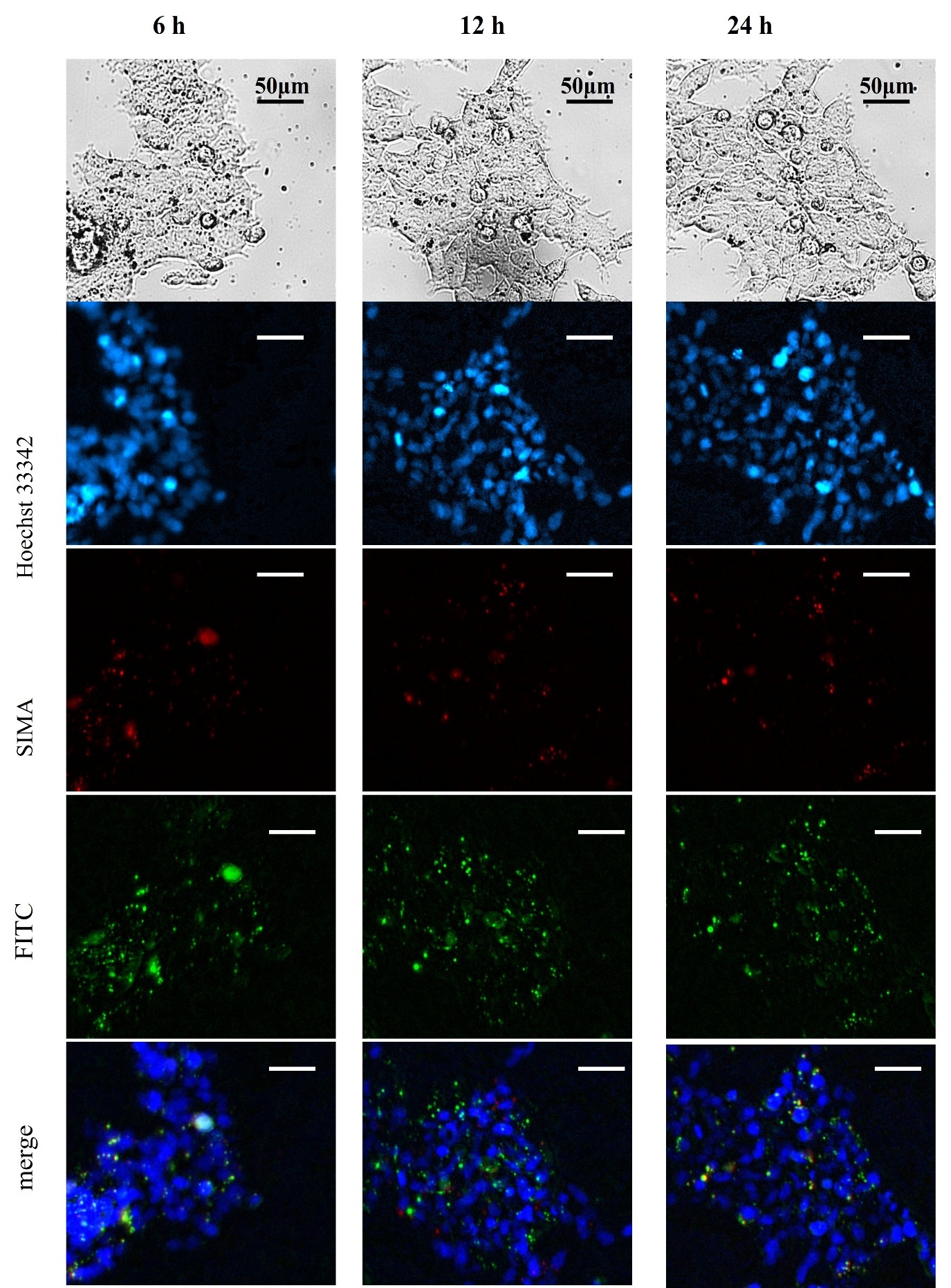


**Figure S5.** Intracellular delivery of SIMA-22AG using HB*pep*-contr: representative light microscopy and fluorescence microscopy images.

**
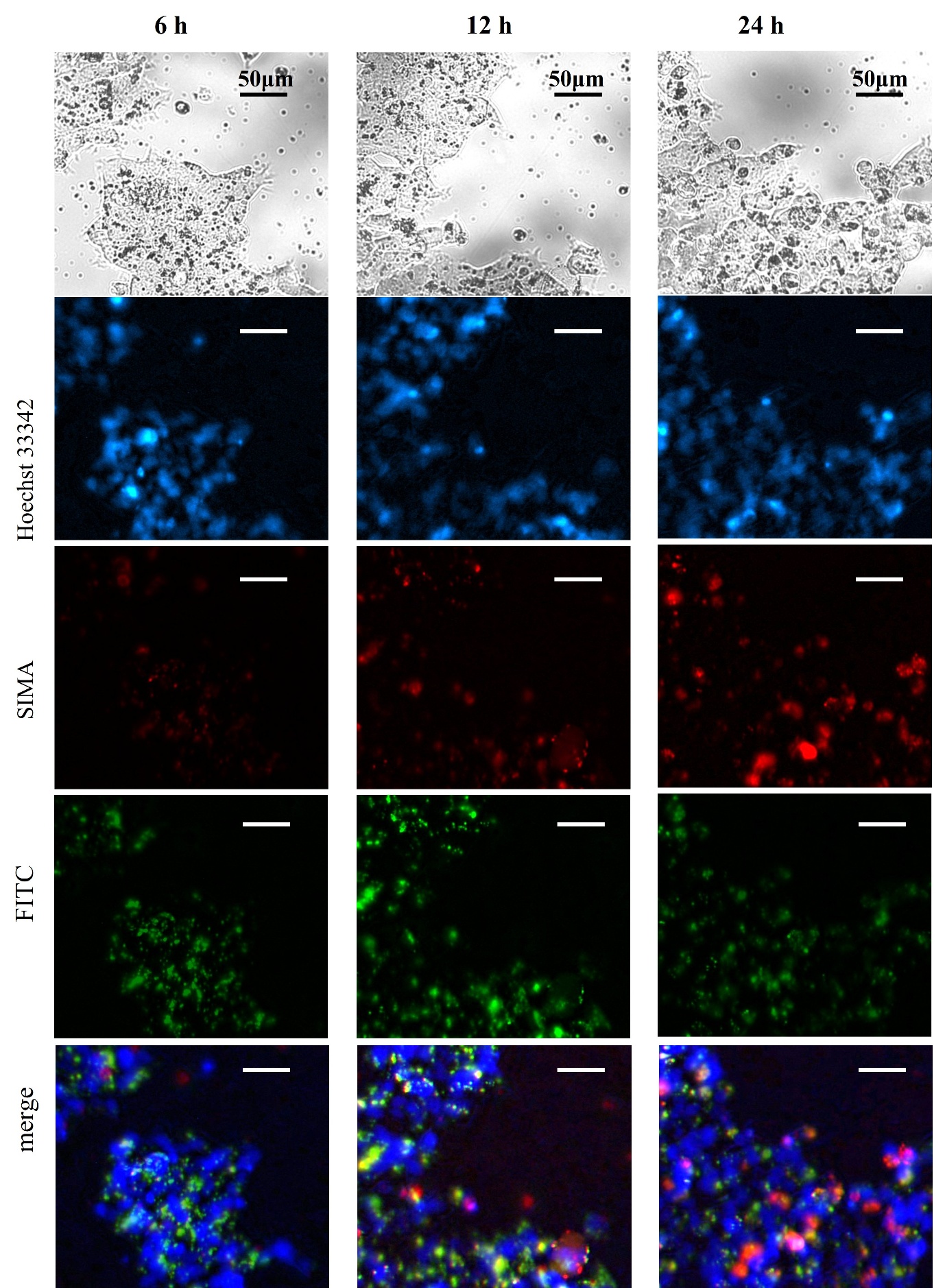
**

**Figure S6.** Intracellular delivery of SIMA-22AG using HB*pep*-ATP-1 representative light microscopy and fluorescence microscopy images.

**
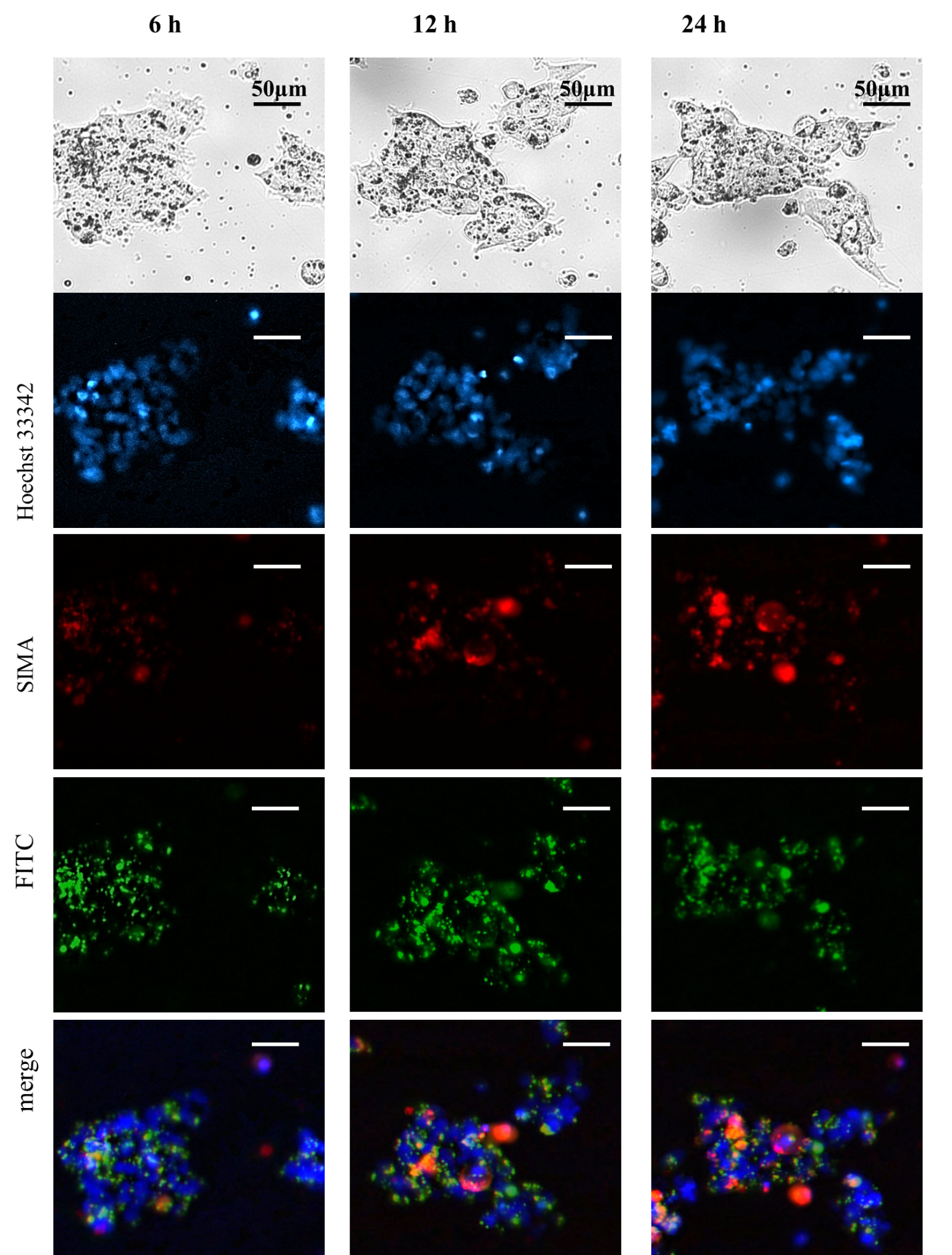
**

**Figure S7.** Intracellular delivery of SIMA-22AG using HB*pep*-ATP-1Nt: representative light microscopy and fluorescence microscopy images.


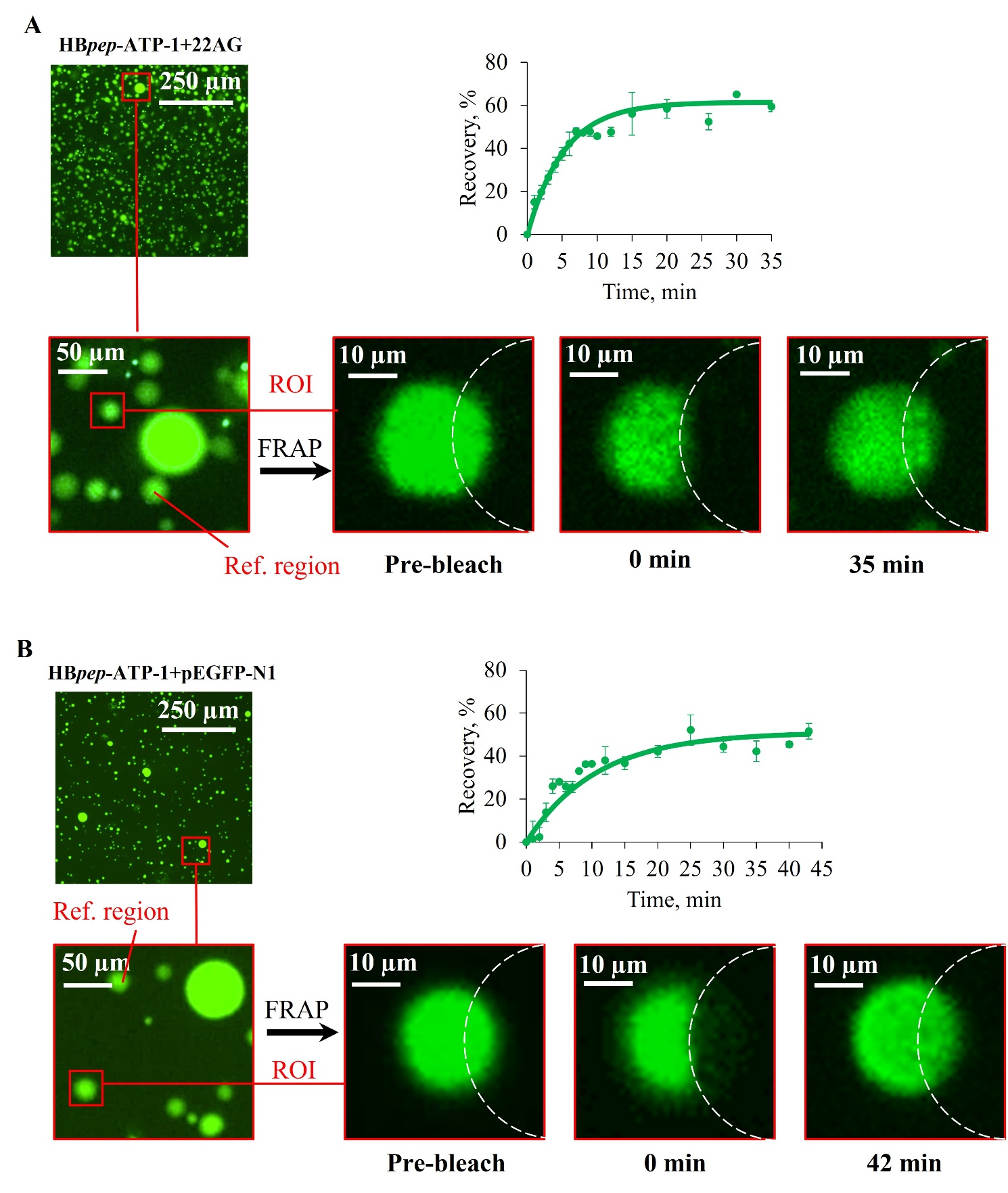


**Figure S8.** The impact of nucleic acid cargo on the density of the coacervates: fluorescence recovery after photobleaching of the HB*pep*-ATP-1 coacervates loaded with 22AG (A) or plasmid DNA (B). The bleached zone is marked by the dashed line.
